# Combining biophysical methods to analyze the disulfide bond in SH2 domain of C-terminal Src kinase

**DOI:** 10.1007/s41048-016-0025-4

**Published:** 2016-07-01

**Authors:** Dongsheng Liu, David Cowburn

**Affiliations:** 1 iHuman Institute, ShanghaiTech University, Shanghai, 201203 China; 2 Department of Biochemistry, Albert Einstein College of Medicine, Bronx, NY 10461 USA

**Keywords:** C-terminal Src kinase, Src homology 2, Disulfide bond, Nuclear magnetic resonance

## Abstract

The Src Homology 2 (SH2) domain is a structurally conserved protein domain that typically binds to a phosphorylated tyrosine in a peptide motif from the target protein. The SH2 domain of C-terminal Src kinase (Csk) contains a single disulfide bond, which is unusual for most SH2 domains. Although the global motion of SH2 domain regulates Csk function, little is known about the relationship between the disulfide bond and binding of the ligand. In this study, we combined X-ray crystallography, solution NMR, and other biophysical methods to reveal the interaction network in Csk. Denaturation studies have shown that disulfide bond contributes significantly to the stability of SH2 domain, and crystal structures of the oxidized and C122S mutant showed minor conformational changes. We further investigated the binding of SH2 domain to a phosphorylated peptide from Csk-binding protein upon reduction and oxidation using both NMR and fluorescence approaches. This work employed NMR, X-ray cryptography, and other biophysical methods to study a disulfide bond in Csk SH2 domain. In addition, this work provides in-depth understanding of the structural dynamics of Csk SH2 domain.

## INTRODUCTION

**Fig. 1:**
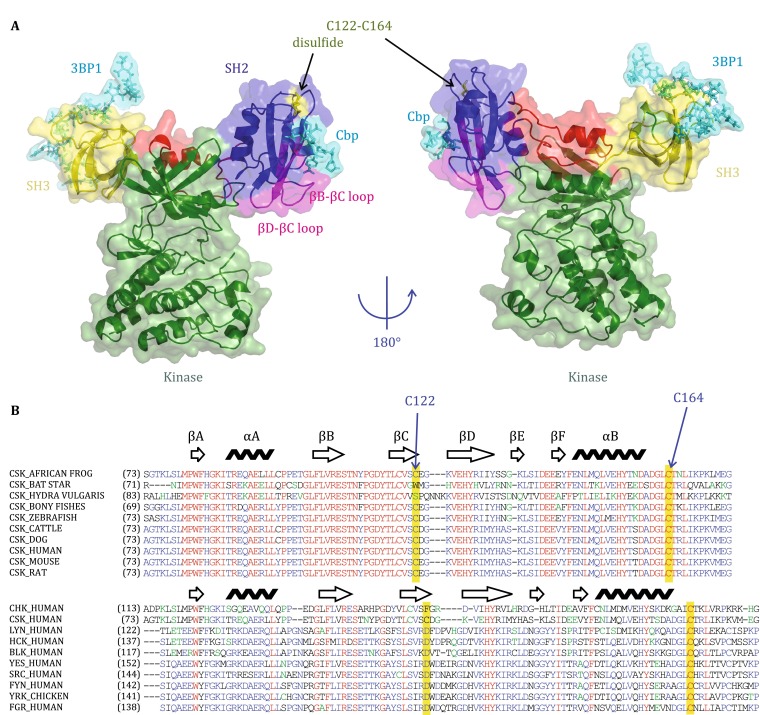
Structure of Csk and sequence alignment of Csk and Src family kinase. **A** Monomeric structure of Csk with bound 3BP1 (to SH3) and CBP peptide (to SH2). The overall structure is plotted with the active Csk1K9A-A. The positions of PEP-3BP1 and CBP peptides are modeled from the structure of 1JEG and 1SPS, respectively. **B** Sequence alignments of Csk family (*upper*) and Csk, Chk and SFKs (*lower*)

C-terminal Src kinase (Csk) and Csk-homologous kinase (Chk) are members of the CSK family of protein tyrosine kinases. These proteins suppress the activity of Src family kinases (SFKs) by selectively phosphorylating the conserved C-terminal tail regulatory tyrosine (Nada et al. 1991, 1993; Chong et al. 2005, 2006). Csk and Chk both contain SH3, SH2, and kinase domains, which are separated by the SH3–SH2 and SH2-kinase linkers (Fig. 1A). The Csk SH2 domain is crucial in stabilizing the kinase domain in the active conformation (Shekhtman et al. 2001; Mikkola and Gahmberg 2010; Grebien et al. 2011). A disulfide bond in the SH2 is suggested to regulate Csk kinase activity (Mills et al. 2007), although the extent is possibly highly assay-specific (Kemble and Sun 2009). The subcellular localization and activity of Csk are also regulated by its SH2 domain (Chong et al. 2005).

Interactions between SH2 domain and tyrosine kinase domain regulate tyrosine kinase signaling networks (Wong et al. 2005; Ia et al. 2010; Mikkola and Gahmberg 2010). With regard to this, Wojcik et al. (2010) for instance described a potent and highly specific FN3 monobody binding to the Abl SH2 domain, which inhibits the kinase (Grebien et al. 2011). The results showed that intramolecular interaction between the SH2 and kinase domains in Bcr-Abl is both necessary and sufficient for the high catalytic activity of the enzyme. Disruption of this interface inhibits the downstream events critical for chronic myelogenous leukemia signaling.

Disulfide bonds are mostly found in secretory proteins and in extracellular domains of membrane proteins. Cytosolic proteins, which contain cysteine residues that are in close proximity to each other, may function as oxidation sensors; when the reductive potential of the cell fails, they oxidize and trigger cellular response mechanisms (Sevier and Kaiser 2002). Mills et al. studied the unique disulfide bond of Csk SH2 that is absent in other known SH2 domains. The kinase activity of full-length Csk is apparently reduced by an order of magnitude upon formation of the disulfide bond in the SH2 domain (Mills et al. 2007). Disulfide bond formation is speculated to exert considerable effects on residues within the kinase domain, most notably within the active-site cleft. Given that most cellular compartments exhibit a reducing environment, disulfide bonds are possibly reduced in the cytosol (Sevier and Kaiser 2002). SH2 sequence alignments from different Csks, Chks, and SFKs show that C122 and C162 are found in most Csks (Fig. 1B).

In this work, we used multiple biophysical methods to investigate the SH2 disulfide bond and the interaction of SH2 with a phosphotyrosine ligand. Comparison of the NMR chemical shift of the oxidized and reduced SH2 domain reveals the major difference that appeared around residue C122 compared with small chemical shift perturbation around residue C164. Denaturation studies have suggested that disulfide bond contributes significantly to the stability of the SH2 domain. Binding affinity of SH2 toward Csk-binding protein (Cbp)-phosphorylated peptide was studied. The reduced and oxidized forms of Csk can both bind with a Cbp peptide, with the reduced SH2 showing a slightly stronger affinity. Crystal structures of both oxidized SH2 and C122S SH2 were solved and refined. Comparison of the crystal structure of the different forms of SH2 suggests that only minor structural changes resulted from the disulfide bond. Analysis of biophysical data of the unusual disulfide bond provided insights into the role of the disulfide bond in SH2 domain.

## RESULTS

### SH3–SH2 linker contributes to the stability of SH2 domain

**Fig. 2:**
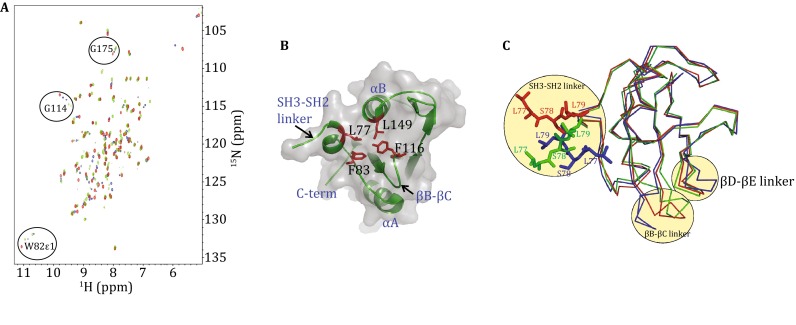
^1^H–^15^N HSQC spectra of different Csk SH2 domains and SH3–SH2 linker region in the crystal structure. **A** SH2 containing M80–A178 (*blue*), P81A SH2 (*green*), and L(Linker)-SH2 containing A73–A178 (*red*). **B** Crystal structure of L-SH2 showing the hydrophobic interaction between SH3–SH2 linker and L149, F83, and Y116 in the SH2 domain. **C** Comparison of SH2 structures in active (*green*), inactive (*red*) full length (PDB ID: 1K9A), and isolated form (*blue*) (PDB ID: 3EAC)

According to Pfam (Punta et al. 2012), the SH2 domain of Csk begins at residue W82. We constructed our first SH2 domain using the residues M80–A178. Two set of peaks were observed in ^1^H–^15^N HSQC spectrum of purified, uniform ^15^N-labeled reduced sample, such as the side chain peak of W82 (Fig. 2A). We postulated that the conformational heterogeneity results from *cis*-*trans* isomerization of the nearby proline P81. We introduced a single point mutation in P81A–SH2 construct and found that the isomerization was not diminished. Therefore, the isomerization probably comes from other residues. Previous studies have suggested that an interaction exists between the SH3–SH2 linker and the SH2 domain (Wong et al. 2005; Mikkola and Gahmberg 2010), so a longer version of SH2 (referred to as L-SH2 and contains A73–A178) was constructed. With the addition of this part of the SH3–SH2 linker into the SH2 domain, isomerization was essentially abolished. This phenomenon made the detailed investigation of the SH2 disulfide bond and ligand binding via NMR practical. The X-ray structure of the oxidized form of L-SH2 (PDB ID: 3EAC) showed that structure of the N-terminal linker area (A73–P81) is well defined (Fig. 2B) with traceable electron density compared with the flexible C-terminus (E174–A178). The crystal structure also revealed that the linker region fold back on the SH2 domain (Fig. 2C). Hydrophobic interactions between M80 and W82 fix the linker position. Additionally, the hydrophobic interactions between L77 in the linker and L149, F83, and Y116 make the linker fold back to the SH2 and thus stabilizing the structure (Fig. 2B). Contact between H84 and M173 was also observed, and this interaction made the N- and C-terminals spatially close to each other. Thus, compared with previous studies, the present work used this longer version of Csk SH2.

### Oxidation and reduction of Csk L-SH2

**Fig. 3:**
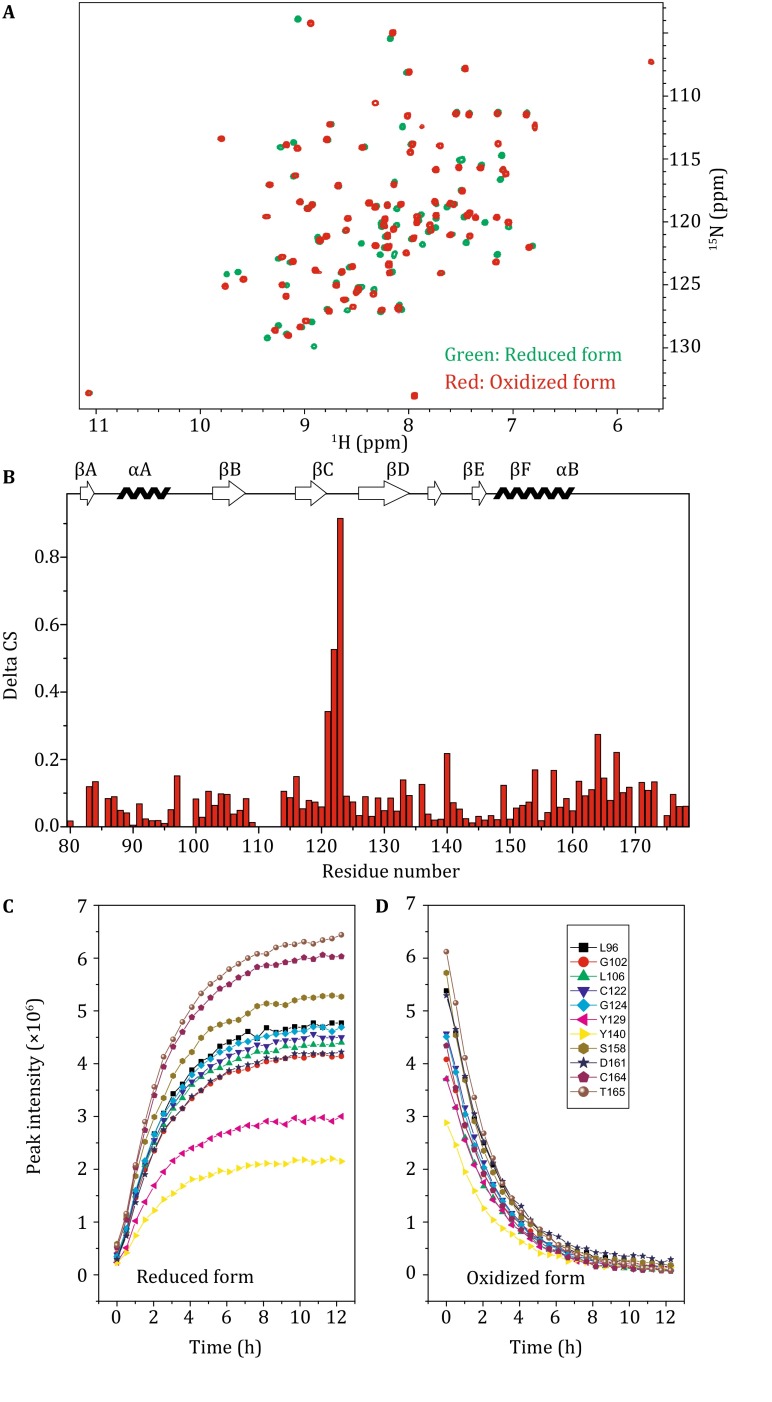
Comparison of the oxidized and reduced form of SH2. **A** Comparison of ^1^H–^15^N HSQC of oxidized (*red*) and reduced (*green*) SH2. **B** Combined chemical shift change of oxidized and reduced form SH2. The difference was calculated as ∆*δ*_tot_ = [(∆*δ*_H_)^2^ + (0.154∆*δ*_N_)^2^]^1/2^. **C** Increase in peak intensity of the reduced form of SH2 upon addition of 10 mmol/L DTT. **D** Reduction in peak intensity of oxidized form of SH2 upon addition of 10 mmol/L DTT. The buffer contains 50 mmol/L Tris–HCl (pH 7.5) with and without 10 mmol/L DTT, 1.0 mmol/L EDTA, 0.01% (*w*/*v*) NaN_3_, 5% D_2_O, and 0.1 mmol/L DSS

During purification of the Csk L-SH2 domain, 50 mmol/L DTT was used to elute the protein from the chitin resin. The L-SH2 was used in reduced form, given that DTT was used to cleave L-SH2 from its intein fusion partner. Following elution of the protein from the ion exchange column, the L-SH2 became a mixture of the reduced and oxidized forms. The fully oxidized form was obtained by exposing the protein to air for several days. Figure 3 shows the comparison of NMR spectra of the reduced and oxidized forms of L-SH2. The oxidized form was obtained from the purified protein dissolved in water at approximately 1 mg/mL and then exposed to air for approximately a week at room temperature and pH 7.2. ^1^H–^15^N HSQC spectra show that the peaks associated with the reduced form disappeared after oxidation, whereas the oxidized peaks appeared. We found that 10 mmol/L DTT is sufficient to reduce the SH2 domain within 10 h, whereas a higher DTT concentration (150 mmol/L) was used in other studies (Mills et al. 2007). Figure 3C shows the oxidized form of SH2 with 10 mmol/L DTT, and the *t*_1/2_ of the reduction reaction was 2.6 ± 0.1 h.

The differences in chemical shifts were measured after assigning both the spectra of the oxidized and reduced forms (Fig. 3B). Most of the changes in chemical shift took place in the residues close to the disulfide bond area, especially near the residue C122. This finding suggested that the C122 area was likely to undergo greater structural changes compared with the C164 residue area.

The NMR chemical shifts of ^13^C^α^ and ^13^C^β^ of cysteine residue can be discriminated between cysteine in its reduced and oxidized states (Sharma and Rajarathnam 2000). The observed C^α^ shifts for oxidized and reduced forms cysteine were 55.5 ± 2.5 and 59.3 ± 3.2 ppm, respectively. The C^β^ chemical shifts of reduced and oxidized cysteine spanned a wider range. The observed C^β^ shifts for the oxidized and reduced cysteine were 40.7 ± 3.8 and 28.4 ± 2.4 ppm, respectively. All of the ^13^C^α^ and ^13^C^β^ chemical shifts of cysteine residues are listed in Table 1 with redox status of the residue indicated in brackets. Residue C119 cannot form a disulfide bond in all of the listed conditions and thus the chemical shift of C119 remained unchanged under either the reduced or oxidized condition. The chemical shift of the ^13^C^α^ of C122 and C164 changed into −5.7 and −0.5 ppm upon the formation of the disulfide bond, respectively. For ^13^C^β^ of C122 and C164, the changes in chemical shift were 16.6 and 13.9 ppm upon the formation of the disulfide bond, respectively. Considerably slight changes in ^13^C chemical shift of these three residues were associated with Cbp peptide binding, suggesting no direct interaction exists between Cbp and the C122–C164 disulfide bond region.

**Table 1:** ^13^C^α^ and ^13^C^β^ chemical shift of all cysteine and cysteine residues in L-SH2 domain

	C119	C122	C164
C^α^(ox)	57.1(S–H)	55.1(S–S)	58.1(S–S)
C^α^(red)	57.1(S–H)	60.8(S–H)	58.6(S–H)
C^α^(red)–Cbp	56.6(S–H)	60.8(S–H)	58.6(S–H)
C^β^(ox)	29.3(S–H)	45.7(S–S)	42.0(S–S)
C^β^(red)	29.4(S–H)	29.1(S–H)	28.1(S–H)
C^β^(red)–Cbp	29.3(S–H)	29.2(S–H)	28.3(S–H)

"Red" or "ox" status of the residue is indicated in bracket

### Disulfide bond formation can significantly enhance the thermal stability of SH2 domain

**Fig. 4:**
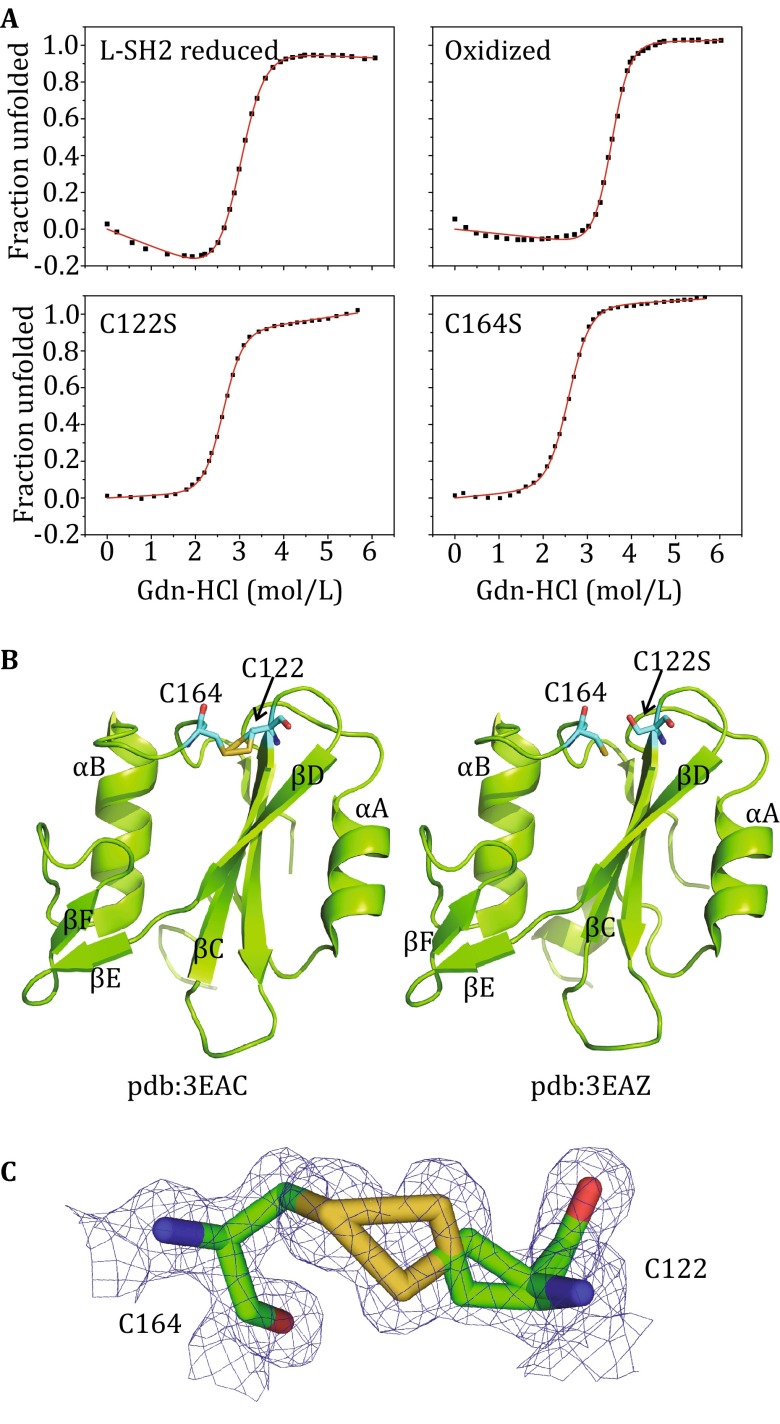
Guanidine hydrochloride denaturation of reduced, oxidized, C122S, and C164S SH2 domain of Csk. **A** The free energies Δ*G*_U,W_^0^ of reduced, oxidized, C122S, and C164S L-SH2 were 27.5 ± 0.7, 42.2 ± 1.6, 29.4 ± 0.9, and 26.3 ± 0.9 kJ/mol, respectively. **B** Comparison of crystal structures of oxidized (*left*) and C122S mutant (*right*) of L-SH2. **C** Electron density map for disulfide bond C122–C164. The density is contoured at the 1 *σ* level

Intrinsic fluorescence of oxidized and reduced form of L-SH2 domain, as well as that of the cysteine mutants C122S and C164S L-SH2, was measured to examine the role of the disulfide bond in the stability of the Csk SH2 domain. The fluorescence spectra of all the mutants were similar to the spectrum of the wild-type domain, which displays a prominent tryptophan maximum emission and excitation at 320 and 288 nm, respectively. Equilibrium denaturation curves exhibit a single, cooperative transition indicative of two-state unfolding. The thermodynamic stability of these oxidized and reduced L-SH2 domains, as well as that of the mutants at equilibrium, were characterized by determining the free energy of unfolding by using guanidine hydrochloride-induced unfolding experiments monitored by tryptophan fluorescence (Fig. 4A). The curves were fitted using the linear extrapolation model to obtain the ∆*G* for unfolding in H_2_O (Santoro and Bolen 1988). The free energies Δ*G*_U,W_^0^ of the reduced, oxidized, C122S and C164S L-SH2 are 27.5 ± 0.7, 42.2 ± 1.6, 29.4 ± 0.9, and 26.3 ± 0.9 kJ/mol, respectively. All of the three forms of L-SH2 without disulfide bond were greatly destabilized compared with L-SH2 containing disulfide bonds with free energy value reductions ranging from 12.8 to 15.9 kJ/mol. Interestingly, substitutions of the C122 and C164 with Ser residues caused a different destabilization. The free energy measurements were also consistent with conservation of the C122 and C164 (Fig. 1B). Formation of the disulfide bond assisted in the packing of αB to the hydrophobic region of the protein consisting of βC and βD (Fig. 4B). The S–S distance of the disulfide bond was 2.0 Å, whereas the O–S distance in the C122S mutant was 3.7 Å (Fig. 4B). As the structure refinement for the oxidized form of L-SH2 progressed, the maps indicated dual conformations for the residue C122 (Fig. 4C), which were successfully modeled as such. Therefore, the high-resolution structures of oxidized L-SH2 reveal dual conformations of disulfide bond that were not observed in the lower-resolution structure of full-length Csk. X-ray crystallography data were also consistent with the NMR observation, in which most of the changes in chemical shift took place in the residues close to C122, and this area was likely to undergo greater structural change than in the C164 residue area (Fig. 3B). Although the side chain direction of C122S residue was slightly different, the overall backbone position was nearly identical to that of the oxidized form and superimposes on the structures of Cα atoms that display a root-mean-square deviation of 0.19 Å.

### Csk SH2 is perturbed upon Cbp peptide binding

**Fig. 5:**
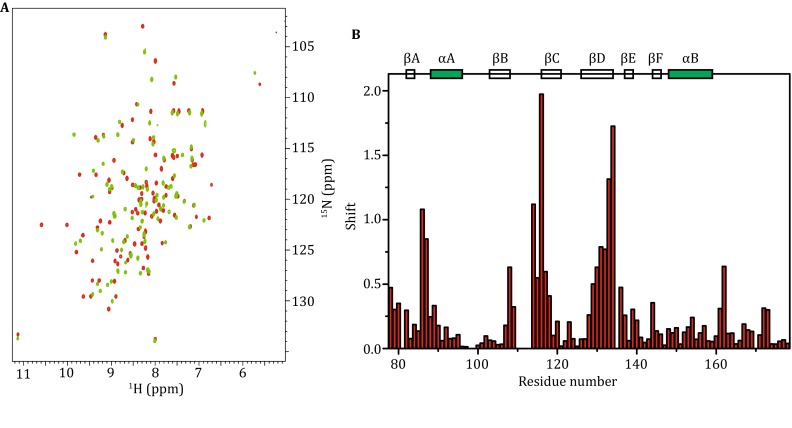
Binding of Cbp peptide with Csk SH2. **A** Overlay of ^1^H–^15^N-HSQC spectra of the Csk SH2 with (*red*) and without (*green*) Cbp peptide. **B** Combined chemical shift change of SH2 with and without Cbp. The difference was calculated as ∆_tot_ = [(∆_H_)^2^ + (0.154∆_N_)^2^]^1/2^

To study the interaction of Csk L-SH2 with Cbp peptide, we performed 3D triple-resonance NMR experiments on L-SH2 samples with and without Cbp peptide. Figure 5 shows the binding of Cbp phosphopeptide with Csk L-SH2. Figure 5A shows the overlay of ^1^H–^15^N HSQC spectra of the Csk L-SH2 with (red) and without (green) Cbp peptide. Figure 5B shows the differences in the chemical shift of the two spectra plotted against the residue number. The perturbed residues on the Csk L-SH2 suggested that βB-βC loop, βD, and βD-βE loop underwent significant conformational changes following Cbp peptide binding. N111 and Y112 were absent from ^1^H–^15^N HSQC spectrum in the absence of ligand; therefore, data on chemical shift perturbation were not available for these two residues. In addition, βA–αA loop residues at the N-terminal underwent smaller chemical shift perturbation upon binding of the phosphopeptide. Comparison of the SH2 structures in putative active full length, inactive full length (Ogawa et al. 2002), and isolated form (PDBs 1K9A-A, 1K9A-C, and 3EAC) also showed that the position of βD–βE and βB–βC loops significantly changed among the active form, inactive form, and isolated SH2. These results suggested that Cbp binding caused a conformational change in the βB–βC, βD, and βD–βE loops and may possibly adjust the conformation in the kinase domain via the contact proposed in a previous study (Ogawa et al. 2002).

### Reduced L-SH2 binds to the phosphorylated tyrosine ligand with slightly stronger affinity than oxidized L-SH2

**Fig. 6:**
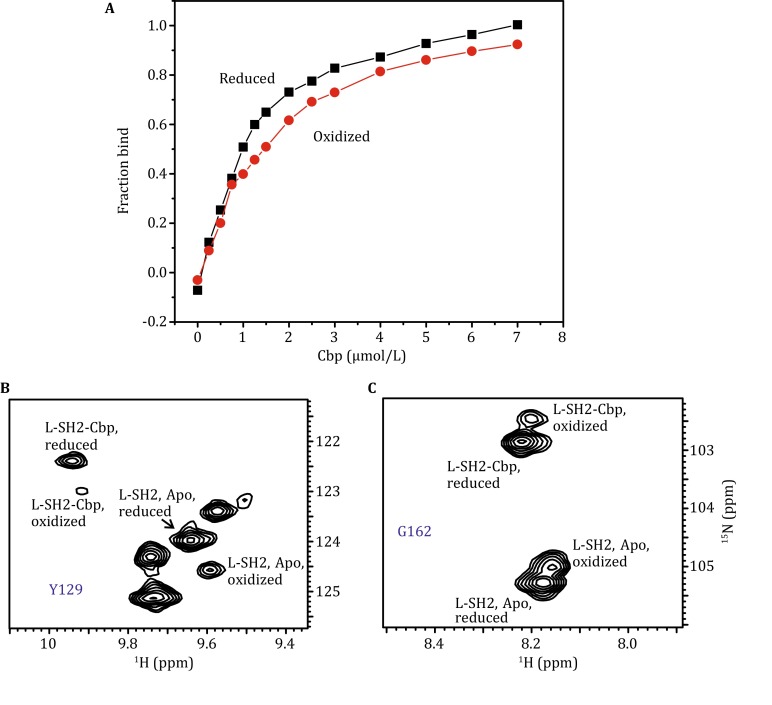
Cbp peptide binding to Csk L-SH2 monitored by fluorescence and NMR. **A** Titration of reduced (*green*) and oxidized (*red*) Csk L-SH2 with Cbp peptide. **B** and **C** HSQC of the mixture of oxidized and reduced form of SH2 (200 μmol/L). Cbp was added up to a final concentration of 100 μmol/L. The residues showing well-resolved four peaks (Y129 and G162 were chosen) can be used in the calculation of relative affinity

To study the interaction between L-SH2 and Cbp, we used a 10-residue peptide with the sequence ISAMpYSSVNK derived from human Cbp protein (Wong et al. 2005). Figure 6A shows the binding of the 10-amino acid peptide ligand with L-SH2. The dissociation constants *K*_d_ extracted from each titration curve were 0.52 ± 0.05 and 0.99 ± 0.08 μmol/L for the reduced and oxidized SH2, respectively, suggesting that the reduced form can bind slightly more efficiently than the oxidized form. To compare the relative binding constant of the reduced and oxidized forms in a single experiment, we used the ^1^H–^15^N HSQC spectrum of the mixture of the oxidized and reduced form of L-SH2 (200 μmol/L). Cbp was titrated with L-SH2 at a final concentration of 100 μmol/L. Well-resolved Y129 and G162 peaks were used to calculate $\frac{{Kd}_{\text{ox}}}{{Kd}_{\text{red}}}$, obtaining 1.5 ± 0.2 and 2.1 ± 0.1, respectively (Fig. 6B, C). The NMR result confirms that the reduced form of L-SH2 binds to Cbp slightly stronger than the oxidized form. The relative ratio of the different forms of peaks observed did not change with time, suggesting that Cbp binding did not alter the dynamics of the formation and breakage of the disulfide bond. Cbp peptide binding of the activation status of kinase domain may be modulated by selecting the reduced form over the oxidized form rather than directly changing the redox status of the Csk-SH2.

## DISCUSSION

Intramolecular interactions between the SH2 domain and tyrosine kinase domain are critical in regulation of catalytic activity. Destabilizing mutations in the SH2 domain and linker region often cause diseases (Filippakopoulos et al. 2009). Studies have suggested that the SH3–SH2 linker interacts with the SH2 domain (Shekhtman et al. 2001; Wong et al. 2005; Mikkola and Gahmberg 2010), and this phenomenon was confirmed by our study. In the crystal structure of the L-SH2, this linker region folds back on the SH2 domain during the hydrophobic interactions, suggesting that the SH3–SH2 domain linker is necessary to stabilize the SH2 domain.

Studies have suggested that the kinase activity of full-length Csk decreases by an order of magnitude upon the formation of the disulfide bond in the distal SH2 domain. Prevention of the reduction of disulfide bond leads to a tenfold reduction in kinase activity, which can be restored upon re-introduction of the reducing agent into the reaction (Mills et al. 2007). Direct and specific inactivation of protein tyrosine kinases in the Src and FGFR families through reversible cysteine oxidation is also observed in alternative procedures, where Csk is mildly activated by DTT (Kemble and Sun 2009). These results suggest that direct redox regulation possibly exists in specific PTKs. In this study, the thermodynamic stability of the oxidized and reduced L-SH2 domains, as well as that of the mutants at equilibrium, was characterized by determining the free energy of unfolding. All of the three forms of SH2 without a disulfide bond were greatly destabilized compared with the SH2 containing a disulfide bond. The disulfide bond formation significantly enhances the thermal stability of SH2 domain and assists in the packing of αB into the hydrophobic region of the protein consisting of βC and βD. The overall backbone position of C122S and the oxidized form in the crystal structure is nearly identical, suggesting that the disulfide stability results from reduction of entropy of the unfolded state.

Csk interacts with Cbp/PAG, which is localized in membrane microdomains enriched with cholesterol, glycosphingolipids, and lipid rafts and is subsequently recruited into the reaction space (Kawabuchi et al. 2000). The interaction occurs between the SH2 domain of Csk and the SFK-phosphorylated Tyr-314 of Cbp (Kawabuchi et al. 2000). In our study, a 10-residue Cbp peptide bound with Csk SH2 with affinity at the µmol/L level, and the chemical shift perturbation pattern was similar to that of the long Cbp peptide (Tanaka et al. 2013), suggesting that the pY314 area was dominant during the interaction between SH2 and Cbp. Compared with the large chemical shift perturbation in L-SH2 caused by Cbp peptide binding, formation of the disulfide bond caused only subtle changes in the L-SH2 domain. In addition, affinity and NMR measurement suggested that the reduced form of L-SH2 can bind with Cbp peptide slightly more efficiently than the oxidized form. Our results also show that Cbp binding did not alter the dynamics of formation and breakage of the disulfide bond; therefore, Cbp peptide binding did not change the redox status of the Csk-SH2. The present work illustrates the combination of NMR, X-ray cryptography, and other biophysical methods in investigating the disulfide bond in SH2 domain. Moreover, this work provides a more complete understanding of the structure and dynamics of SH2 domain. However, mechanistic details linking the disulfide bond to the regulation of Csk kinase activity remain elusive until further structural study of the disulfide bond in the context of full-length Csk.

## MATERIALS AND METHODS

### Protein expression and purification

The human Csk SH2 domain genes containing the residues A73–A178 were amplified and cloned into the expression vector pTWIN1 (New England Biolabs) as described previously (Liu et al. 2009). The plasmid was transformed into a BL21 (DE3) RIL component cell (Stratagene, 230245). The cells were grown to mid-log phase and induced with 0.5 mmol/L IPTG at 25 °C overnight. After centrifugation, the cells were resuspended in 35 mL of Buffer A (50 mmol/L Tris–HCl, pH 7.5, 200 mmol/L NaCl) and passed through a French pressure cell twice. The cell lysate was centrifuged at 12,000*g* for 20 min at 4 °C. The clarified cell extract was loaded into 6 mL of chitin beads. The cleavage of the intein-tag was induced by equilibrating the chitin beads with 50 mmol/L DTT, 50 mmol/L KH_2_PO_4_–K_2_HPO_4_ at pH 7.2 for 24 h. The target protein was eluted and further purified by a Mono Q column on Äkta system (GE Healthcare). The Cbp peptide (ISAMpYSSVNK) was synthesized by GenScript (Piscataway, NJ) using standard solid-phase peptide synthesis methods and then resuspended in purified water before use.

### NMR spectroscopy

All NMR experiments were performed at 298 K on 800 MHz spectrometers equipped with triple-resonance cryoprobes. Protein solutions were prepared under the following buffer conditions: 0.8 mmol/L protein in 50 mmol/L Tris–HCl (pH 7.5), 1.0 mmol/L EDTA, 0.01% (*w*/*v*) NaN_3_, 5% D_2_O, and 0.1 mmol/L DSS (4,4-dimethyl-4-silapentane-1-sulfonate). DTT (10 mmol/L) was added whenever necessary. In the 3D triple-resonance experiments, HNCA, HNCO, HNCACB, and CBCA(CO)NH were collected for backbone resonance assignment. The ^1^H chemical shifts were referenced to internal DSS. The ^13^C and ^15^N chemical shifts were referenced indirectly using the ^1^H/^13^C or ^1^H/^15^N frequency ratios of the zero point: 0.101329118 (^15^N) and 0.251449530 (^13^C) (Live et al. 1984; Wishart et al. 1995). The combined change in chemical shift of a particular residue upon ligation with the kinase domain was calculated as ∆*δ* = [(∆*δ*_H_)^2^ + (0.154∆*δ*_N_)^2^]^1/2^, where ∆*δ*_H_ and ∆*δ*_N_ correspond to the changes in amide proton and nitrogen chemical shift, respectively. The weight factor for ^1^H and ^15^N is determined from the ratio of the average variances of the amide nitrogen and proton chemical shifts observed for the 20 common amino acid residues in proteins deposited in the BioMagResBank (Mulder et al. 1999). For those residues showing well-resolved four peaks (reduced-bound, reduced-apo, oxidized-bound, and oxidized-apo), the relative binding affinity was calculated as follows:

$$ \frac{{Kd}_{\text{ox}}}{{Kd}_{\text{red}}} = \frac{{\frac{{\left[ {{\text{SH2}}_{\text{ox}} } \right]\left[ {\text{Cbp}} \right]}}{{\left[ {{\text{SH2}}_{\text{ox}} \cdot {\text{Cbp}}} \right]}}}}{{\frac{{\left[ {{\text{SH2}}_{\text{red}} } \right]\left[ {\text{Cbp}} \right]}}{{\left[ {{\text{SH2}}_{\text{red}} \cdot {\text{Cbp}}} \right]}}}} = \frac{{\left[ {{\text{SH2}}_{\text{ox}} } \right]\left[ {{\text{SH2}}_{\text{red}} \cdot {\text{Cbp}}} \right]}}{{\left[ {{\text{SH2}}_{\text{red}} } \right]\left[ {{\text{SH2}}_{\text{ox}} \cdot {\text{Cbp}}} \right]}}. $$

Peak intensity represents the relative concentration of each fraction.

### Crystallization, data collection, and structural analysis

The oxidized form of the Csk L-SH2 domain or the C122S mutant was crystallized using the hanging drop vapor diffusion method. Protein solution (1 μL, 15 mg/mL) was mixed with reservoir solution [1 μL, 100 mmol/L Bis-Tris (pH 7.3), 22% PEG 4000] and incubated in reservoir solution (1 mL) at 25 °C. Within one day, crystals typically grew as rod-shaped structures with dimensions 300 μm × 20 μm × 20 μm. X-ray diffraction data were collected, integrated, and scaled using HKL2000 (Otwinowski and Minor 1997). Structure-factor amplitudes were calculated using TRUNCATE (Bailey 1994). Data on diffraction were consistent with the orthorhombic space group P_212121_, with unit cell dimensions as follows: *a* = 37.1 Å, *b* = 48.0 Å, *c* = 50.0 Å and *a* = 37.5 Å, *b* = 48.1 Å, *c* = 49.9 Å for the oxidized and C122S mutant, respectively. Unless otherwise stated, all programs used for structural and crystallographic analyses were located within the CCP4 interface of the CCP4 suite (Bailey 1994). The structure of Csk L-SH2 or C122S mutant domain was solved using the molecular replacement method. Initial phases were obtained using MOLREP, and the co-ordinates of the SH2 domain with Protein Data Bank (PDB) entry code 1K9A were used as search models (Ogawa et al. 2002). Manual model rebuilding was performed in Coot (Emsley and Cowtan 2004), and maximum likelihood refinement was performed using REFMAC5 (Murshudov et al. 1997). Ordered water molecules were initially added into the model using Coot and eventually added manually. The program PROCHECK (Laskowski et al. 1993) was used to assess the quality of the final structures. Data collection and refinement statistics are shown in Table 2. Co-ordinates were deposited in PDB under accession codes 3EAC (oxidized) and 3EAZ (C122S mutant).

**Table 2:** Data collection and refinement statistics

	Csk L-SH2 oxidized	Csk L-SH2 C122S
Cell constant [*a*, *b*, *c* (Å)]	37.1, 48.0, 50.0	37.5, 48.2, 49.9
Resolution from map calculation (Å)	29.45–1.37	25.45–1.31
Space group	P212121	P212121
Number of reflections	18,298	22,354
Completeness (%)	99.7	99.8
Refinement [*R* (%)/*R*_free_ (%)]	0.209/0.217	0.196/0.226
Water molecules	106	118
Ramachandran plot (favor/allowed/disallowed)	81.8/18.1/0	87.6/12.4/0

### Fluorescence measurement

Fluorescence titrations were performed at 25 °C in 3 mL of 50 mmol/L Tris-HCl (pH 7.5) using excitation and emission wavelengths of 288 nm and 320 nm, respectively. The 10-residue synthetic phosphopeptide corresponding to the specific SH2-binding site in Cbp was titrated with the L-SH2 sample. The typically adopted protein concentration is 5 μmol/L. For the denaturation experiments, ultrapure guanidine hydrochloride (Sigma S0933) was used. Proteins were exposed to guanidine hydrochloride concentrations ranging from 0 to 6 mol/L at 0.1–0.2 mol/L steps. The concentrations of guanidine hydrochloride of the solutions were determined by measuring the index of refraction and by using the following equation: *d*/*d*_0_ = 1 + 0.2710 *W* + 0.0330 *W* ^2^ (Tanford et al. 1966), where *W* is the weight fraction of guanidine hydrochloride in the solution, *d* is the density of the solution, and *d*_0_ is the density of water. Reversibility of the guanidine hydrochloride-induced unfolding reaction and the time necessary to re-establish equilibrium were determined by diluting a concentrated protein solution containing 6 mol/L guanidine hydrochloride into the buffer and by monitoring the fluorescence spectrum of the protein as a function of time. The signals were normalized to the fraction of unfolded species using the standard relation *F*_unf_ = (*I* − *I*_N_)/(*I*_U_ − *I*_N_), where N and U stand for the fluorescence intensity of the native and fully unfolded species, respectively. *F*_unf_ values were calculated from the linear extrapolation of the pre- and post-unfolding baselines. The unfolding free energy and *m* values in the relationship were obtained from the fitting of the denaturation data into a two-state model using the standard equation for all four L-SH2 protein samples. The apparent free energy of unfolding was determined according to the linear equation Δ*G*_U,W_^0^ = Δ*G*_U,D_^0^ + *m*[D],  where Δ*G*_U,W_^0^ is the apparent free energy of unfolding in water, Δ*G*_U,D_^0^ is the apparent free energy of unfolding at a denaturant concentration [D], and *m* is the coefficient expressing the dependence of the free energy of unfolding on the denaturant concentration.

### Accession numbers

PDB number: 3EAC (oxidized) and 3EAZ (C122S mutant). The backbone chemical shifts of SH2 with and without Cbp peptide were deposited in BMRB with accession numbers 7141 and 7140, respectively.

## Abbreviations

Csk: C-terminal Src kinase
SH2: Src homology 2
Cbp: Csk-binding protein
SFK: Src family kinase
HSQC: Heteronuclear single quantum correlation

## References

[CR1] Bailey S (1994). The ccp4 suite: programs for protein crystallography. Acta Crystallogr D.

[CR2] Chong YP, Mulhern TD, Cheng HC (2005). C-terminal Src kinase (CSK) and CSK-homologous kinase (CHK)—endogenous negative regulators of Src-family protein kinases. Growth Factors.

[CR3] Chong YP, Chan AS, Chan KC, Williamson NA, Lerner EC, Smithgall TE, Bjorge JD, Fujita DJ, Purcell AW, Scholz G, Mulhern TD, Cheng HC (2006). C-terminal Src kinase-homologous kinase (CHK), a unique inhibitor inactivating multiple active conformations of Src family tyrosine kinases. J Biol Chem.

[CR4] Emsley P, Cowtan K (2004). Coot: model-building tools for molecular graphics. Acta Crystallogr D Biol Crystallogr.

[CR5] Filippakopoulos P, Muller S, Knapp S (2009). SH2 domains: modulators of nonreceptor tyrosine kinase activity. Curr Opin Struct Biol.

[CR6] Grebien F, Hantschel O, Wojcik J, Kaupe I, Kovacic B, Wyrzucki AM, Gish GD, Cerny-Reiterer S, Koide A, Beug H, Pawson T, Valent P, Koide S, Superti-Furga G (2011). Targeting the SH2-kinase interface in Bcr-Abl inhibits leukemogenesis. Cell.

[CR7] Ia KK, Mills RD, Hossain MI, Chan K-C, Jarasrassamee B, Jorissen RN, Cheng H-C (2010). Structural elements and allosteric mechanisms governing regulation and catalysis of CSK-family kinases and their inhibition of Src-family kinases. Growth Factors.

[CR8] Kawabuchi M, Satomi Y, Takao T, Shimonishi Y, Nada S, Nagai K, Tarakhovsky A, Okada M (2000). Transmembrane phosphoprotein Cbp regulates the activities of Src-family tyrosine kinases. Nature.

[CR9] Kemble DJ, Sun G (2009). Direct and specific inactivation of protein tyrosine kinases in the Src and FGFR families by reversible cysteine oxidation. Proc Natl Acad Sci USA.

[CR10] Laskowski RA, Macarthur MW, Moss DS, Thornton JM (1993). PROCHECK: a program to check the stereochemical quality of protein structures. J Appl Crystallogr.

[CR11] Liu D, Xu R, Cowburn D (2009). Segmental isotopic labeling of proteins for nuclear magnetic resonance. Methods Enzymol.

[CR12] Live DH, Davis DG, Agosta WC, Cowburn D (1984). Long range hydrogen bond mediated effects in peptides: nitrogen-15 NMR study of gramicidin S in water and organic solvents. J Am Chem Soc.

[CR13] Mikkola ET, Gahmberg CG (2010). Hydrophobic interaction between the SH2 domain and the kinase domain is required for the activation of Csk. J Mol Biol.

[CR14] Mills JE, Whitford PC, Shaffer J, Onuchic JN, Adams JA, Jennings PA (2007). A novel disulfide bond in the SH2 domain of the C-terminal Src kinase controls catalytic activity. J Mol Biol.

[CR15] Mulder FA, Schipper D, Bott R, Boelens R (1999). Altered flexibility in the substrate-binding site of related native and engineered high-alkaline Bacillus subtilisins. J Mol Biol.

[CR16] Murshudov GN, Vagin AA, Dodson EJ (1997). Refinement of macromolecular structures by the maximum-likelihood method. Acta Crystallogr D Biol Crystallogr.

[CR17] Nada S, Okada M, MacAuley A, Cooper JA, Nakagawa H (1991). Cloning of a complementary DNA for a protein-tyrosine kinase that specifically phosphorylates a negative regulatory site of p60c-src. Nature.

[CR18] Nada S, Yagi T, Takeda H, Tokunaga T, Nakagawa H, Ikawa Y, Okada M, Aizawa S (1993). Constitutive activation of Src family kinases in mouse embryos that lack Csk. Cell.

[CR19] Ogawa A, Takayama Y, Sakai H, Chong KT, Takeuchi S, Nakagawa A, Nada S, Okada M, Tsukihara T (2002). Structure of the carboxyl-terminal Src kinase, Csk. J Biol Chem.

[CR20] Otwinowski Z, Minor W (1997). Processing of X-ray diffraction data collected in oscillation mode. Method Enzymol.

[CR21] Punta M, Coggill PC, Eberhardt RY, Mistry J, Tate J, Boursnell C, Pang N, Forslund K, Ceric G, Clements J, Heger A, Holm L, Sonnhammer EL, Eddy SR, Bateman A, Finn RD (2012). The Pfam protein families database. Nucleic Acids Res.

[CR22] Santoro MM, Bolen DW (1988). Unfolding free energy changes determined by the linear extrapolation method. 1. Unfolding of phenylmethanesulfonyl alpha-chymotrypsin using different denaturants. Biochemistry.

[CR23] Sevier CS, Kaiser CA (2002). Formation and transfer of disulphide bonds in living cells. Nat Rev Mol Cell Biol.

[CR24] Sharma D, Rajarathnam K (2000). C-13 NMR chemical shifts can predict disulfide bond formation. J Biomol NMR.

[CR25] Shekhtman A, Ghose R, Wang D, Cole PA, Cowburn D (2001). Novel mechanism of regulation of the non-receptor protein tyrosine kinase Csk: insights from NMR mapping studies and site-directed mutagenesis. J Mol Biol.

[CR26] Stevens R, Stevens L, Price NC (1983). The stabilities of various thiol compounds used in protein purifications. Biochem Educ.

[CR27] Tanaka H, Akagi K, Oneyama C, Tanaka M, Sasaki Y, Kanou T, Lee YH, Yokogawa D, Dobenecker MW, Nakagawa A, Okada M, Ikegami T (2013). Identification of a new interaction mode between the Src homology 2 (SH2) domain of C-terminal Src kinase (Csk) and Csk-binding protein (Cbp)/phosphoprotein associated with glycosphingolipid microdomains. J Biol Chem.

[CR28] Tanford C, Kawahara K, Lapanje S (1966). Proteins in 6-M guanidine hydrochloride. Demonstration of random coil behavior. J Biol Chem.

[CR29] Wishart DS, Bigam CG, Holm A, Hodges RS, Sykes BD (1995). H-1, C-13 and N-15 random coil NMR chemical-shifts of the common amino-acids.1. investigations of nearest-neighbor effects. J Biomol NMR.

[CR30] Wojcik J, Hantschel O, Grebien F, Kaupe I, Bennett KL, Barkinge J, Jones RB, Koide A, Superti-Furga G, Koide S (2010). A potent and highly specific FN3 monobody inhibitor of the Abl SH2 domain. Nat Struct Mol Biol.

[CR31] Wong L, Lieser SA, Miyashita O, Miller M, Tasken K, Onuchic JN, Adams JA, Woods VL, Jennings PA (2005). Coupled motions in the SH2 and kinase domains of Csk control Src phosphorylation. J Mol Biol.

